# Authentication of Bulbus Fritillariae Cirrhosae by RAPD-Derived DNA Markers

**DOI:** 10.3390/molecules19033450

**Published:** 2014-03-20

**Authors:** Gui-Zhong Xin, Yin-Ching Lam, Maitinuer Maiwulanjiang, Gallant K. L. Chan, Kevin Yue Zhu, Wai-Lun Tang, Tina Ting-Xia Dong, Zi-Qi Shi, Ping Li, Karl W. K. Tsim

**Affiliations:** 1 Division of Life Science and Center for Chinese Medicine, The Hong Kong University of Science and Technology, Clear Water Bay Road, Hong Kong, China; E-Mails: xinguizhong1984@126.com (G.-Z.X.); kellylam@ust.hk (Y.-C.L.); mavlanjan@hotmail.com (M.M.); gallant@ust.hk (G.K.L.C.); nzyzy808@163.com (K.Y.Z.); roytwl@ust.hk (W.-L.T.); botina@ust.hk (T.T.-X.D.); 2 State Key Laboratory of Natural Medicines, China Pharmaceutical University, No. 24 Tongjia Lane, Nanjing 210009, China; E-Mail: shiziqi47@126.com

**Keywords:** Bulbus Fritillariae Cirrhosae, RAPD, SCAR, herbal drug authentication

## Abstract

Bulbus Fritillariae is the most commonly used antitussive herb in China. Eleven species of *Fritillaria* are recorded as Bulbus Fritillariae in the Chinese Pharmacopoeia. Bulbus Fritillariae Cirrhosae is a group of six *Fritillaria* species with higher efficiency and lower toxicity derived mainly from wild sources. Because of their higher market price, five other *Fritillaria* species are often sold deceptively as Bulbus Fritillariae Cirrhosae in the herbal market. To ensure the efficacy and safety of medicinal herbs, the authentication of botanical resources is the first step in quality control. Here, a DNA based identification method was developed to authenticate the commercial sources of Bulbus Fritillariae Cirrhosae. A putative DNA marker (0.65 kb) specific for Bulbus Fritillariae Cirrhosae was identified using the Random Amplified Polymorphic DNA (RAPD) technique. A DNA marker representing a Sequence Characterized Amplified Region (SCAR) was developed from a RAPD amplicon. The SCAR marker was successfully applied to differentiate Bulbus Fritillariae Cirrhosae from different species of *Fritillaria*. Additionally, the SCAR marker was also useful in identifying the commercial samples of Bulbus Fritillariae Cirrhosae. Our results indicated that the RAPD-SCAR method was rapid, accurate and applicable in identifying Bulbus Fritillariae Cirrhosae at the DNA level.

## 1. Introduction

The bulbs of several *Fritillaria* species (Lilliaceae), named “Bei-mu” in Chinese, have been used in China as antitussive, antiasthmatic and expectorant herbs for more than 2,000 years [1,2]. There are eleven species of *Fritillaria* recorded as the drug Bulbus Fritillariae in the Chinese Pharmacopoeia [3], which are divided into five groups: (1) Bulbus Fritillariae Cirrhosae (Chuanbeimu); (2) Bulbus Fritillariae Pallidiflorae (Yibeimu); (3) Bulbus Fritillariae Thunbergii (Zhebeimu); (4) Bulbus Fritillariae Hupehensis (Hubeibeimu); and (5) Bulbus Fritillariae Ussuriensis (Pingbeimu). Among these five groups, Bulbus Fritillariae Cirrhosae is considered to be the top-grade herb for use as an antitussive and expectorant drug.

According to the Chinese Pharmacopoeia, Bulbus Fritillariae Cirrhosae is the dried bulb of *Fritillaria cirrhosa* D. Don, *F. unibracteata* Hsiao et K. C. Hsia, *F. przewalskii* Maxim., *F. delavayi* Franch., *F. taipaiensis* P. Y. Li or *F. unibracteata* Hsia et K. C. Hsia var. *wabuensis* CS. Y. Tang et S. C. Yue, Z. D. Liu, S. Wang et S. C. Chen [3]. Bulbus Fritillariae Cirrhosae is rare and expensive, because most of these *Fritillaria* species are only found wild and distributed at an altitude of 3000–5000 m. Studies have suggested that Bulbus Fritillariae Cirrhosae has better therapeutic effects and lower toxicity, as compared to other species of Bulbus Fritillariae [4,5]. Consequently, some producers deceptively sell other species of *Fritillaria* as Bulbus Fritillariae Cirrhosae in the market. Thus, the authentication of species is of primary importance for ensuring efficacy and safety of the herb, and therefore, the development of method to effectively authenticate Bulbus Fritillariae Cirrhosae would be of great value.

Authentication based on morphological, microscopic or phytochemical characteristics is not reliable enough [6,7,8] because the plant can vary due to different practices of planting, harvesting, storage and manufacturing. The use of DNA identification methods is far more reliable; because DNA sequences do not vary easily. DNA sequencing methods already have been used to identify bulb of *Fritillaria* species [9,10]. Based on sequence analysis of the 5S-rRNA spacer region from different species of *Fritillaria*, a unique sequence was found in *F. cirrhosa.* However, this method cannot be used to identify bulbs of other species in the Bulbus Fritillariae Cirrhosae. Li *et al*. developed a polymerase chain reaction-restriction fragment length polymorphism (PCR-RFLP) method for the simultaneous identification of Bulbus Fritillariae Cirrhosae [11,12]. However, the PCR-RFLP was a two-step reaction method and required high quality DNA samples [10]. Unfortunately, it is always difficult to obtain high quality DNA from raw Chinese herbs. In this study, a SCAR marker was designed from RAPD polymorphisms for rapid and easy identification of Bulbus Fritillariae Cirrhosae. This DNA marker was further used to authenticate the commercial samples of Bulbus Fritillariae Cirrhosae. The reliability of our results was validated using the PCR-RFLP method. Overall, the RAPD-derived SCAR method proved to be a quick, simple, accurate and effective mean in identifying Bulbus Fritillariae Cirrhosae at DNA level.

## 2. Results and Discussion

### 2.1. Results

#### 2.1.1. Identification of RAPD Marker for Bulbus Fritillariae Cirrhosae

**Figure 1:**
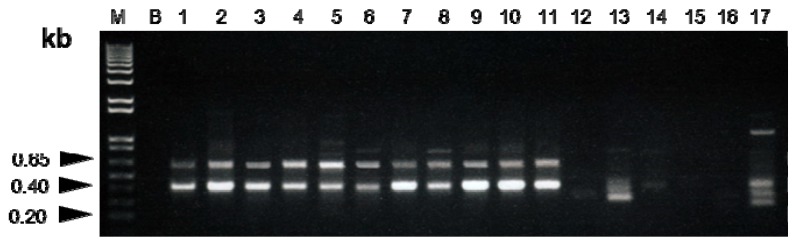
RAPD profiles of *Fritillaria* species amplified with primer S158. The specific primers revealed species-specific patterns for *Fritillaria* species of Bulbus Fritillariae Cirrhosae. M: 1 kb DNA ladder; B: Blank (nuclease-free distilled water) Lane 1–11: species of Bulbus Fritillariae Cirrhosae: *Fritillaria cirrhosa* (1–2), *F. unibracteata* (3–4), *F. przewalskii* (5–6), *F. delavayi* (7–8), *F. taipaiensis* (9–10), and *F. unibracteata* Hsia et K. C. Hsia var. *wabuensis* (11) Lane 12–17: Other *Fritillaria* species: *F. hupehensis* (12), *F. ussuriensis* (13), *F. thunbergii* (14), *F. pallidiflora* (15), *F. walujewii* (16), and *F. puqiensis* (17).

High molecular weight genomic DNA was isolated from dry *Fritillaria* bulbs, which yielded 50–300 ng of DNA per 50 mg of bulbs. An absorbance (A260/A280) ratio of 1.6–1.8 suggested an insignificant level of contaminating proteins and/or polysaccharides. Seven of the twenty-four RAPD primers, for which variability and applicability were tested in preliminary experiments, were chosen for further analysis. With these primers all specimens showed highly repeatable patterns with the finally chosen protocol and reaction conditions (data not shown). However, only the primer S158 consistently amplified a single, intense band of ~0.65 kb for the six species of Bulbus Fritillariae Cirrhosae, which was absent in other six *Fritillaria* species (Figure 1). This band, named as CBM08, was putatively designated as Bulbus Fritillariae Cirrhosae specific marker. Bulbs of different *Fritillaria* species were listed in Table 1.

**Table 1:** Plant species, origins and groups for medicinal purposes.

Groups	Species	NO. of Indiv.	Origin
Chuanbeimu	*Fritillaria cirrhosa*	2	Kangdin County, Sichuan
	*F. unibracteata*	2	Hongyuan County, Sichuan
	*F. przewalskii*	2	Min County, Gansu
	*F. delavayi*	2	Gongjue County, Tibet
	*F. taipaiensis*	2	Chongqing
	*F. unibracteata* Hsia et K. C. Hsia var. *wabuensis*	1	Mao County, Sichuan
Yibeimu	*F. pallidiflora*	1	Yili County, Xinjiang
	*F. walujewii*	1	Tacheng County, Xinjiang
Zhebeimu	*F. thunbergii*	1	Ningbo, Zhejiang
Hubeibeimu	*F. hupehensis*	1	Yichang, Hubei
Pingbeimu	*F. ussuriensis*	1	Tonghua, Jilin
Puqibeimu	*F. puqiensis*	1	Puqi County, Hubei

#### 2.1.2. Cloning and Sequencing of the RAPD Marker

**Figure 2:**
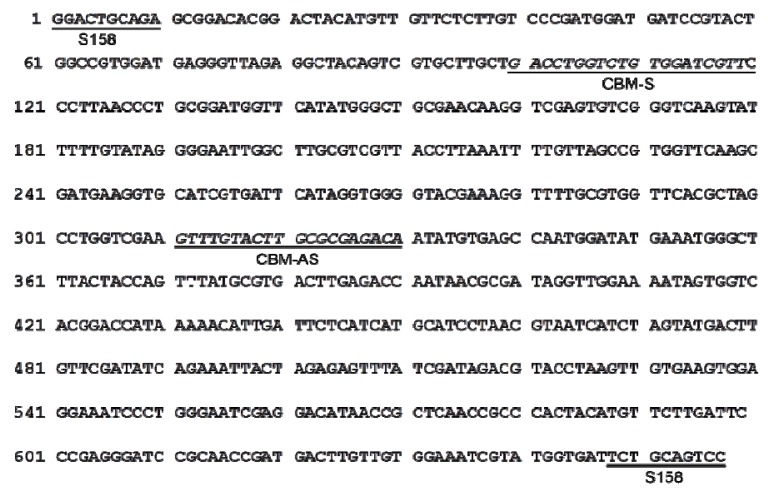
Nucleotide sequence of the RAPD amplicon CBM08; the RAPD primers (a pair of S158) and SCAR primers (CBM-S and CBM-AS) were indicated.

The ~0.65 kb CBM08 band was isolated and sequenced. The first ten nucleotides of the sequence matched with the corresponding RAPD primer used. This result clearly showed that the cloned fragment was derived from the amplified RAPD product. The length of the CBM08 marker sequence obtained was 657 bp with 47% G + C content (A: 161; C: 129; G: 182; T: 186) (Figure 2). BLAST results revealed that the sequence showed partial homology with known plant nucleotide sequences at different similarity levels. Considerable similarity was found with the sequence of *Fritillaria imperialis* (Scor: 45.4 bits, and E-value: 5E-18).

#### 2.1.3. Amplification using SCAR Primers

**Figure 3:**
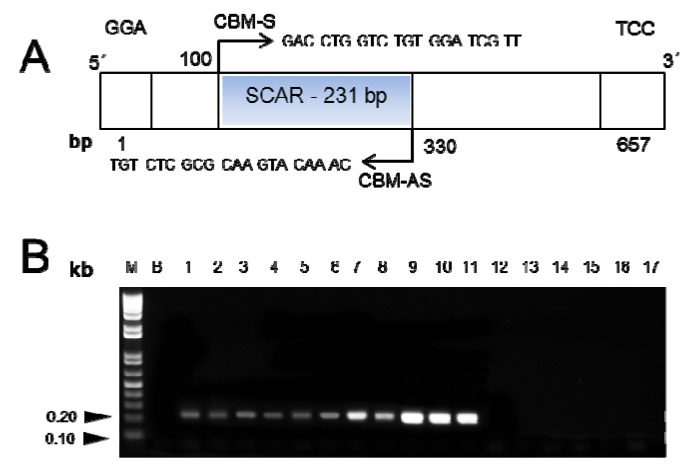
(**A**) Schematic diagram of RAPD and SCAR amplifying region. (**B**) PCR amplification of *Fritillariae* species using SCAR (CBM-S and CBM-AS) primers. The specific band (231 bp) was only detected in species of Bulbus Fritillariae Cirrhosae. M: 1kb DNA ladder; B: Blank (nuclease-free distilled water). Lane 1-11: species of *Bulbus Fritillariae Cirrhosae*: *Fritillaria cirrhosa* (1–2), *F. unibracteata* (3–4), *F. przewalskii* (5–6), *F. delavayi* (7–8), *F. taipaiensis* (9–10), and *F. unibracteata* Hsia et K. C. Hsia var. *wabuensis* (11). Lanes 12–17: Other *Fritillaria* species: *F. hupehensis* (12), *F. ussuriensis* (13), *F. thunbergii* (14), *F. pallidiflora* (15), *F. walujewii* (16), and *F. Puqiensis* (17).

**Figure 4:**
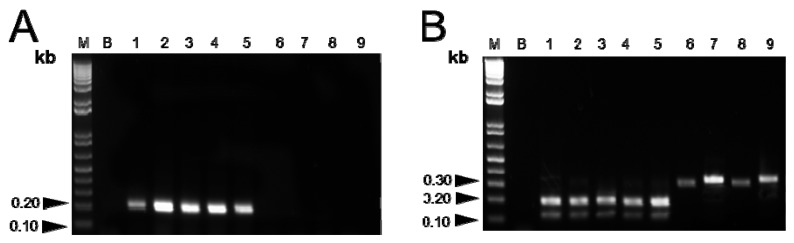
(**A**) PCR amplification of nine commercially available crude drugs of Bulbus Fritillariae Cirrhosae using SCAR marker amplification (CBM-S and CBM-AS); A specific band (231 bp) for Bulbus Fritillariae Cirrhosae was detected in samples 1–5. (**B**) PCR-RFLP analysis of Bulbus Fritillariae Cirrhosae validated results obtained using the SCAR marker. The detail protocol was displayed in part *3.8*. The five samples generated two fragments between 100–200 bp after being digested by *SmaI*, confirming that they were Bulbus Fritillariae Cirrhosae (B).

The designed SCAR primer pair (Table 2), CBM-S and CBM-AS, was used to amplify genomic DNA from the twelve *Fritillaria* species (Figure 2 and Figure 3A). A single and distinct band of 231 bp was obtained in DNA isolated from all the six *Fritillaria* species of Bulbus Fritillariae Cirrhosae, while no specific amplification was observed for any of the other six *Fritillaria* species (Figure 3B). The SCAR primers were further used to amplify DNA from nine commercial samples of *Fritillaria* bulbs. A sharp and reproducible band (231 bp) was observed for five samples (Figure 4A), each purchased from different drug shops in Hong Kong, which verified these samples were authentic Bulbus Fritillariae Cirrhosae.

**Table 2:** Specific SCAR primer of Bulbus Fritillariae Cirrhosae devised from sequenced RAPD amplicon CBM08.

RADM Primer	SCAR Primer	Number of base pairs (bp)	Sequence (5'-3')	G + C Content (%)	Annealing temperature
S158	CBM-S	20	GAC CTG GTC TGT GGA TCG TT	55	59 °C
	CBM-AS	20	TGT CTC GCG CAA GTA CAA AC	50	

In contrast, no specific band was observed for the other four *Fritillaria* bulbs, which indicated that these samples were not Bulbus Fritillariae Cirrhosae (Figure 4A).

#### 2.1.4. Validation of Results

In order to validate the reliability of our RAPD-SCAR method, the authentication of commercial samples was validated using the previously reported PCR-RFLP method. Two fragments (~190 and ~118 bp) were obtained following the digestion of the PCR products from five samples (*i.e.*, samples 1–5) digested by *SmaI* (Figure 4B), which indicated that these samples were authentic Bulbus Fritillariae Cirrhosae. The fragments from other four commercial samples (samples 6–9) could not be digested by *SmaI* (Figure 4B), which indicated that they were not Bulbus Fritillariae Cirrhosae. The corroborative results of these two methods were totally coincidental. The basic features of each method are compared in Table 3.

**Table 3:** The characters of PCR-RFLP and RAPD-SCAR methods.

Column heading	PCR-RFLP	RAPD-SCAR
Specificity on *Fritillariae* *Cirrhosae Bulbus*	Yes	Yes
Require high quality DNA	Yes	No
Require prior DNA sequence Information	Yes	No
Experimental steps	2	1
Experimental duration	6–8 h	2–4 h

### 2.2. Discussion

This work aimed to improve the authentication process of Bulbus Fritillariae Cirrhosae by using a RAPD-derived molecular method. RAPD analysis can reveal a high degree of polymorphism, and which does not require prior DNA sequence information of the species. More important, this method is simple and straightforward, which makes it suitable for application in the Traditional Chinese Medicine industries. Many researchers have explored the use of RAPD markers for authentication of traditional Chinese medicines like *Panax* [13], *Cordyceps sinensis* [14] and *Atractylodes* [15]. In our RAPD analysis, significant genetic polymorphism was observed among the *Fritillaria* species surveyed. As recorded in the 2010 Chinese Pharmacopoeia [3], there are six *Fritillaria* species used as source material of Bulbus Fritillariae Cirrhosae. In our study, the random primer S158 yielded a ~0.65 kb amplicon specific for Bulbus Fritillariae Cirrhosae. We selected the monomorphic band (CBM08) for SCAR marker development. In SCAR, pairs of oligonucleotide primers specific to the sequence of polymorphic bands can be used to amplify the characterized regions from genomic DNA. Based on the sequence of CBM08, SCAR primers (CBM-S, CBM-AS) were designed to amplify a small region of 231 bp.

Utility of CBM-S and CBM-AS was tested for the identification of Bulbus Fritillariae Cirrhosae from the commercial samples of dried bulbs. The SCAR primers amplified an expected 231 bp DNA fragment in five of tested samples, but not in other four samples. The reliability of the above method was demonstrated by the previously developed PCR-RFLP method. Consistent results demonstrated that the novel SCAR marker was able to distinguish Bulbus Fritillariae Cirrhosae from other *Fritillaria* species. The SCAR marker has advantages for large scale analyses because of low cost, high reproducibility, and ease of use without elaborate electrophoresis. We expect that our SCAR marker will complement other molecular methods used to identify Bulbus Fritillariae Cirrhosae.

## 3. Experimental

### 3.1. Materials

Bulbs of different *Fritillaria* species were collected in China. Plant specimens were identified by the authors and voucher specimens were deposited in the Center for Chinese Medicine of Hong Kong University of Science and Technology. In addition, nine commercial samples of *Fritillaria* bulbs (1–9) were collected in Hong Kong herbal markets.

### 3.2. DNA Extraction

For DNA extraction, the dried Bulbs were ground into a fine powder. The extraction was performed with a commercial kit (DNeasy^®^Plant Mini Kit, Qiagen, Hilden, Germany) according to the manufacturer’s protocol. The DNA quantification was done with a NanoDrop^®^ ND-1000 spectrophotometer (DiaMed China Limited, Hong Kong, China).

### 3.3. RAPD Analysis

PCR for amplification conditions were optimized to increase the reproducibility of the banding pattern. With the optimized reaction conditions and PCR protocol, the patterns were highly reproducible in multiple experiments. The RAPD was carried out with 5 μL of genomic DNA (~50 ng) in a 50 μL reaction containing 10× PCR buffer (with Mg^2+^ at a 1X conc. of 1.5 mM and contains loading dye), 0.5 mM dNTP, 400 nM primer and 1.5 U of Taq Polymerase (KAPA Taq DNA Polymerase with dye, KAPA Biosystems, Woburn, MA, USA). The PCR was carried out with a GeneAmp^®^ PCR System 9700 (Applied Biosystems; Foster City, CA, USA). The cycling conditions consisted of an initial 5 min at 94 °C followed by 1 min denaturing for at 94 °C, 1 min annealing at 36 °C, and 1 min elongation at 72 °C repeated 40 cycles and with 10 min of final extension at 72 °C. An aliquot (5 μL) of the amplification product was separated on a 1.2% agarose gel and detected under UV-light after staining in ethidium bromide.

### 3.4. Screening Strategy and Identification of Specific RAPD

Twenty-four random primers were screened for their ability to produce a marker specific to Bulbus Fritillariae Cirrhosae. Amplification of DNA from the twelve *Fritillaria* species including six *Fritillaria* species of Bulbus Fritillariae Cirrhosae was carried out using a single primer. The amplicon was monomorphic in all the *Fritillaria* species of Bulbus Fritillariae Cirrhosae but absent in other six *Fritillaria* species.

### 3.5. Cloning of the RAPD Amplicon

The putative marker amplified by the random primer S158 was excised from 1.2% agarose gel with sterile gel slicer and purified using QIAquick Genei Gel Extraction kit (Qiagen). It was then used as a template for its re-amplification using the same primer. TA cloning strategy was employed using the Invitrogen TA Cloning system (Invitrogen, Carlsbad, CA, USA). The standard reaction consisted of 10× ligation buffer (1 μL), PCR Vector (2 μL, 50 ng), PCR product (6 μL), and T4 DNA ligase (1 μL). The reaction mixture was mixed by pipetting, and incubated at 4 °C overnight. Transformation was carried out using high efficiency competent cells (DH-5α strain of *Escherichia coli*) following protocol for transformation by calcium chloride as described by Sambrook *et al*. [16]. Ten distinct white colonies were picked up from the LB-ampicillin plate. Then, the recombinant plasmid DNA from *E. coli* was isolated using Qiagen’s QIAprep^®^ Mini prep kit following the manufacturer’s directions. Confirmation of the clones was done by amplifying the DNA using M13 and T7 universal primers.

### 3.6. Sequencing, SCAR Primer Designing

After the purified RAPD amplicon was cloned, both ends of the recombinant plasmid were sequenced on an ABI 3700 automated sequencer (Applied Biosystems). Homology searches were performed within Genebank’s non-redundant database using the BLAST algorithm [17]. Based on the sequenced RAPD amplicon, a pair of SCAR oligonucleotide primer (CBM-S and CBM-AS), which could amplify approximately 231 bp fragment in Bulbus Fritillariae Cirrhosae was designed. CBM-S was designed as the forward primer and CBM-AS as the reverse primer. The sequences were custom synthesized from Life Technologies Limited, Hong Kong.

### 3.7. Amplification of the Genomic Region

The SCAR primers pair was used for PCR amplifications of genomic DNA from the twelve *Fritillaria* species (including six *Fritillaria* species of Bulbus Fritillariae Cirrhosae). Thermal cycling conditions for amplification using SCAR primers were optimized as: 94 °C for 5 min; 40 cycles at 94 °C for 1 min, 59 °C for 1 min., and 72 °C for 1 min; and a final extension at 72 °C for 10 min. PCR amplification for authentication of commercial samples was done by using the SCAR primer pair with the above-mentioned thermal cycling conditions.

### 3.8. Validation of the Authentication Result

The result of authentication of commercial samples was validated by the PCR-RFLP method. The identification of Bulbus Fritillariae Cirrhosae by PCR-RFLP was carried out using the protocol described by Li *et al.* [11]. Briefly, the genomic DNA of nine commercial samples was first amplified with previously reported primers ITS-P1 (5'-CGTAACAAGGTTTCCGTAGGTGAA-3') and ITS-P3 (5'-GCTACGTTCTTCATCGAT-3'). Thermal cycling conditions for amplification were: 95 °C for 4 min; 30 cycles at 95 °C for 30 s, 58 °C for 30 s, and 72 °C for 30 s; and a final extension at 72 °C for 5 min. Then, the PCR products were digested using the incision enzyme *SmaI*. As reported [11], there was a *SmaI* digestion site in Bulbus Fritillariae Cirrhosae DNA sequence (CCCGGG), but not in other Bulbus Fritillariae (CTCGGG). Digestions with *SmaI* (10 U/μL, Roche, Basel, Switzerland) were performed in a total volume of 20 μL containing 6 μL PCR products, 5U of *SmaI* enzyme, 2 μL 10× digestion buffer as recommended by the manufacturer, and 11.5 μL of PCR H_2_O; The incubation was at 37 °C for 1.5 h.

## 4. Conclusions

The developed RAPD-SCAR method effectively distinguished the Bulbus Fritillariae Cirrhosae from other species of *Fritillaria*. It was also successfully applied to identifying commercial samples of dried bulbs, which was then analyzed by PCR-RFLP method for verification. The results demonstrated that the RAPD-SCAR method was simple, accurate and reliable in identifying Bulbus Fritillariae Cirrhosae at the DNA level. Our study suggests that the use of RAPD-derived DNA markers to authenticate Bulbus Fritillariae Cirrhosae could be helpful to ensuring the quality and clinical efficacy of *Fritillaria* bulbs. Additionally, this method could facilitate the testing and certification of other Chinese herbal materials in the plant pharmaceutical industry.
